# High Prevalence of Extended-Spectrum Beta-Lactamases in *Escherichia coli* Strains Collected From Strictly Defined Community-Acquired Urinary Tract Infections in Adults in China: A Multicenter Prospective Clinical Microbiological and Molecular Study

**DOI:** 10.3389/fmicb.2021.663033

**Published:** 2021-07-07

**Authors:** Peiyao Jia, Ying Zhu, Xue Li, Timothy Kudinha, Yang Yang, Ge Zhang, Jingjia Zhang, Yingchun Xu, Qiwen Yang

**Affiliations:** ^1^Department of Clinical Laboratory, State Key Laboratory of Complex Severe and Rare Diseases, Peking Union Medical College Hospital, Chinese Academy of Medical Sciences and Peking Union Medical College, Beijing, China; ^2^Graduate School, Peking Union Medical College, Chinese Academy of Medical Sciences, Beijing, China; ^3^Department of Clinical Laboratory, Beijing Anzhen Hospital, Capital Medical University, Beijing, China; ^4^School of Biomedical Sciences, Charles Sturt University, Orange, NSW, Australia; ^5^NSW Health Pathology, Regional and Rural, Orange Hospital, Orange, NSW, Australia

**Keywords:** community-acquired urinary tract infections, *Escherichia coli*, extended-spectrum beta-lactamase, antibiotic resistance, empirical treatment, CTX-M

## Abstract

**Objective:**

The objective of the study was to investigate the antimicrobial susceptibility and extended-spectrum beta-lactamase (ESBL) positive rates of *Escherichia coli* from community-acquired urinary tract infections (CA-UTIs) in Chinese hospitals.

**Materials and Methods:**

A total of 809 *E. coli* isolates from CA-UTIs in 10 hospitals (5 tertiary and 5 secondary hospitals) from different regions in China were collected during the period 2016–2017 according to the strict inclusion criteria. Antimicrobial susceptibility testing was carried out by standard broth microdilution method. Isolates were categorized as ESBL-positive, ESBL-negative, and ESBL-uncertain groups according to the CLSI recommended phenotypic screening method. ESBL and AmpC genes were amplified and sequenced on ESBL-positive and ESBL-uncertain isolates.

**Results:**

The antimicrobial agents with susceptibility rates of greater than 95% included imipenem (99.9%), colistin (99.6%), ertapenem (98.9%), amikacin (98.3%), cefmetazole (97.9%), nitrofurantoin (96%), and fosfomycin (95.4%). However, susceptibilities to cephalosporins (varying from 58.6% to 74.9%) and levofloxacin (48.8%) were relatively low. In the phenotypic detection of ESBLs, ESBL-positive isolates made up 38.07% of *E. coli* strains isolated from CA-UTIs, while 2.97% were ESBL-uncertain. Antimicrobial susceptibilities of imipenem, cefmetazole, colistin, ertapenem, amikacin, and nitrofurantoin against ESBL-producing *E. coli* strains were greater than 90%. The percentage of ESBL-producing strains was higher in male (53.6%) than in female patients (35.2%) (*p* < *0.001*). CTX-M-14 (31.8%) was the major CTX-M variant in the ESBL-producing *E. coli*, followed by CTX-M-55 (23.4%), CTX-M-15 (17.5%), and CTX-M-27 (13.3%). The prevalence of carbapenem-resistant *E. coli* among CA-UTI isolates was 0.25% (2/809).

**Conclusion:**

Our study indicated high prevalence of ESBL in *E. coli* strains from strictly defined community-acquired urinary tract infections in adults in China. Imipenem, colistin, ertapenem, amikacin, and nitrofurantoin were the most active antimicrobials against ESBL-positive *E. coli* isolates. *bla*_*CTX–M–*__14_ is the predominant *esbl* gene in ESBL-producing and ESBL-uncertain strains. Our study indicated that the use of cephalosporins and fluoroquinolone needs to be restricted for empirical treatment of CA-UTIs in China.

## Introduction

*Escherichia coli (E. coli)* is a common pathogen of community-acquired infections such as intra-abdominal infection, urinary tract infection (UTI), and pelvic inflammatory disease. UTI is one of the most common bacterial infectious diseases of humans. Over 85% of community-acquired urinary tract infections (CA-UTIs) have been attributed to *E. coli* infection (Espínola et al., 2011). Increasing trend in extended-spectrum beta-lactamase (ESBL) rates was seen among isolates from CA-UTIs in many regions; the antimicrobial resistance surveillance program in China showed that the proportion of ESBL-producing *E. coli* in community-acquired infections ranges from 45.2 to 68.2% (Yun et al., 2014; Fupin et al., 2017), in Canada, from 9.1 to 14.1%, and in the United States, from 6.5% in 2010 to 16.0% in 2014 (Lob et al., 2016). In some European countries, the prevalence of ESBL-producing *E. coli* isolated from CA-UTIs is currently lower than 5% (van Driel et al., 2019; Richelsen et al., 2020; Larramendy et al., 2021), but can reach up to 23.6% in Spanish, 38.2% in Turkey, and 34.6% in Iran (Arana et al., 2017; Koksal et al., 2017; Naziri et al., 2020). Most of the discrepancy in ESBL prevalence rates between studies might be related to geographical difference. However, the inclusion and exclusion criteria of isolates is also an important factor, which may over- or underestimate the ESBL rates or antimicrobial resistance in community-acquired infections. Studying the antimicrobial resistance patterns of *E. coli* in real community-acquired infections is important not only for understanding the resistance status but also for choosing the most appropriate empirical antimicrobial therapy for CA-UTIs (Lob et al., 2015). The real ESBL rate and molecular epidemiology in CA-UTIs in China is unclear.

The aim of this study was to investigate the real ESBL status and antimicrobial susceptibility of *E. coli* isolates strictly collected from CA-UTIs in China.

## Materials and Methods

### Clinical Isolates

**FIGURE 1:**
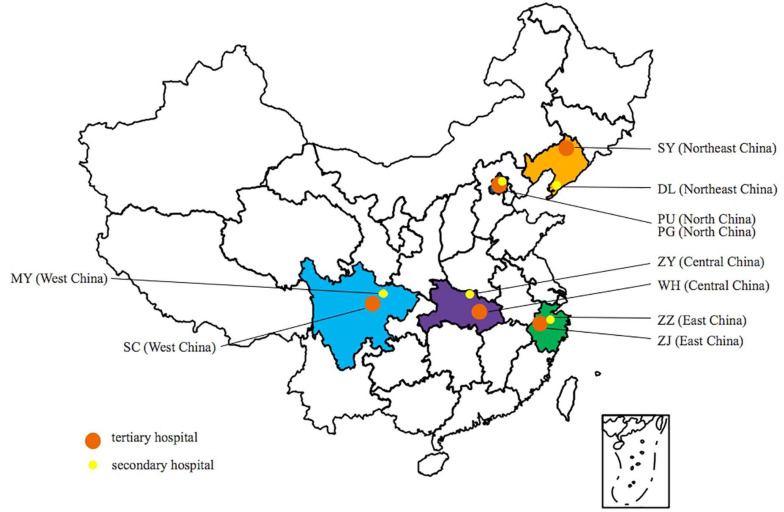
Geographical distribution of hospitals participating in the study. PU, Peking Union Medical College Hospital; PG, Beijing Pinggu Hospital; SY, Shengjing Hospital of China Medical University; DL, Dalian Hospital; ZZ, Zhuji People’s Hospital of Zhejiang Province; ZJ, The First Affiliated Hospital College of Medical Zhejiang University; WH, Tongji Hospital; ZY, First Peoples Hospital of Zaoyang; SC, Sichuan Provincial People’s Hospital; and MY, Sichuan Science City Hospital.

During the period 2016–2017, a total of 809 *E. coli* isolates from CA-UTIs were consecutively collected from 10 hospitals located in the following regions of China: northeastern (121 isolates), northern (179 isolates), central (172 isolates), western (159 isolates), and eastern (178 isolates). The specific geographical distribution is shown in Figure 1. The 10 hospitals included 5 tertiary hospitals (Peking Union Medical College Hospital; Sichuan Provincial People’s Hospital; Tongji Hospital, Tongji Medical College Huazhong University of Science and Technology; The First Affiliated Hospital, College of Medicine, Zhejiang University; and Shengjing Hospital of China Medical University) and 5 secondary hospitals located in the same provinces as the 5 tertiary hospitals (Beijing Pinggu Hospital; Sichuan Science City Hospital; Zaoyang First People’s Hospital; Zhuji People’s Hospital of Zhejiang Province; and Dalian Hospital, Shengjing Hospital of China Medical University).

Isolates were strictly chosen by using the following inclusion and exclusion criteria to ensure all cases were community-acquired patients.

#### Inclusion Criteria

(1) All *E. coli* isolates were cultured from urines of adult UTI patients (>18 years old) from outpatient clinic/emergency department or admitted to a hospital in less than 48 h. (2) Isolates were cultured from uncomplicated UTI, such as acute cystitis, acute pyelonephritis, with evidence support of clinical symptoms, urine routine test, and/or imaging examination. (3) Isolates were infection-related pathogens cultured from qualified urine specimens with bacteria quantification of > 10^5^ CFU/ml.

#### Exclusion Criteria

Isolates from patients with the following conditions were excluded; (1) patients with invasive devices (such as various venous catheters, urethral catheters, intubations, and artificial implants, etc.); (2) immunocompromised patients (e.g., patients who had received glucocorticoid, radiotherapy, and chemotherapy within 6 months); (3) patients with a history of surgery, hemodialysis/abdominal dialysis, hospitalization, and community clinic/health center hospitalization within the previous 1 year; (4) using broad-spectrum antibiotics within 3 months prior to infection; (5) patients with chronic urinary tract infections (previously isolated from the same type of specimen); (6) isolates implicated in healthcare-associated infections and recurrent UTIs (recurrences of uncomplicated and/or complicated UTIs, with a frequency of at least three UTIs/year or two UTIs in the last 6 months); (7) environmental samples or cultures for infection control purposes; and (8) duplicate isolates (the same species from the same patient).

### Antimicrobial Susceptibility Test Method

Minimum inhibitory concentration determination for all antimicrobial agents, except fosfomycin, was performed using the microdilution broth method as per Clinical and Laboratory Standards Institute (CLSI) guidelines (CLSI, 2020). MICs of fosfomycin were determined by agar dilution method (25 μg/ml of glucose-6-phosphate was added in Mueller–Hinton agar). Seventeen antimicrobial agents were analyzed, including cefazolin (CZO), ceftriaxone (CRO), ceftazidime (CAZ), cefepime (FEP), cefoperazone/sulbactam (CSL, 2:1), imipenem (IPM), ertapenem (ETP), amikacin (AMK), levofloxacin (LVX), cefmetazole (CMZ), trimethoprim/sulfamethoxazole (SXT, 1:19), colistin (COL), fosfomycin (FOS), cefotaxime (CTX), cefotaxime/clavulanic acid (CTC), ceftazidime/clavulanic acid (CCV), and nitrofurantoin (NIT). For each batch of MIC testing, reference strains *E. coli* ATCC 25922 and *P. aeruginosa* ATCC 27853 were used as quality control organisms.

### Phenotypic Detection of Extended-Spectrum β-Lactamases

Phenotypic identification of ESBL in *E. coli* was carried out by CLSI-recommended methods (CLSI, 2020). If cefotaxime or ceftazidime MIC of an isolate was ≥ 2 μg/ml each, the MICs of cefotaxime + clavulanic acid (4 μg/ml) or ceftazidime + clavulanic acid (4 μg/ml) were comparatively determined. ESBL production was defined as a greater than or equal to eightfold decrease in MICs for cefotaxime or ceftazidime when tested in combination with clavulanic acid, compared with their MICs without clavulanic acid. ESBL-negative isolates were defined as isolates with cefotaxime or ceftazidime MICs of ≤ 1 μg/ml. ESBL-uncertain isolates were defined as isolates with cefotaxime or ceftazidime MICs of ≥ 2 μg/ml, but did not exhibit a greater than or equal to eightfold decrease in MICs for cefotaxime or ceftazidime after a combination with clavulanic acid, compared with their MICs alone.

### Characterization of Antibiotic Resistance Genes

The main *esbl* and *ampC* genes, including *bla*_*TEM*_, *bla*_*SHV*_, *bla*_*CTX–M–*__1_ group, *bla*_*CTX–M–*__2_ group, *bla*_*CTX–M–*__9_ group, *bla*_*DHA*_, *bla*_*CMY*_, and *bla*_*ACT*_, were determined using polymerase chain (PCR) reaction method on the strains with ESBL-producing and ESBL-uncertain phenotype. The positive amplicons were sequenced and aligned by blastn web^1^. Primers used in this study are listed in Table 1.

**TABLE 1:** Polymerase chain reaction (PCR) primers used for detecting antibiotic resistance genes.

Target		Primer sequences (5′ to 3′)	Annealing temp. (°C)	Fragment size (bp)	References
TEM	Forward	ATAAAATTCTTGAAGACGAAA	55	1,079	Yang et al., 2015
	Reverse	GACAGTTAGCAATGCTTAATCA			
SHV	Forward	CCGGGTTATTCTTATTTGTCGCT	56	928	Lee et al., 2006
	Reverse	TAGCGTTGCCAGTGCTCG			
CTX-M-1 group	Forward	CGTCACGCTGTTGTTAGGAA	56	823	Yang et al., 2015
	Reverse	ACCGTCGGTGACGATTTTAG			
CTX-M-2 group	Forward	ATGATGACTCAGAGCATTCG	65	832	Yang et al., 2015
	Reverse	TCCCGACGGCTTTCCGCCTT			
CTX-M-9 group	Forward	AAAAATGATTGAAAGGTGGT	56	1,242	Yang et al., 2015
	Reverse	GTGAAGAAGGTGTTGCTGAC			
DHA-1	Forward	CTGATGAAAAAATCGTTATC	56	1,141	Giakkoupi et al., 2006
	Reverse	ATTCCAGTGCACTCAAAATA			
CMY	Forward	TGTCAACACGGTGCAAATCA	56	1,346	Armand-Lefèvre et al., 2003
	Reverse	AGCAACGACGGGCAAAATG			
ACT-1	Forward	CGAACGAATCATTATTCAGCACCG	56	1,518	Reisbig and Hanson, 2002
	Reverse	CGGCAATGTTTACTACACAGCG			

### Statistical Analysis

The results of antimicrobial susceptibility testing were analyzed by the WHONET5.6 program. Ninety-five percent confidence intervals were calculated using the adjusted Wald method; comparison of ESBL rates between tertiary and secondary hospital, in demographic characteristics and clinical features, was assessed using Chi-square test. Analyses were performed using SPSS version 25.0 (IBM Corporation), and *p*-values < 0.05 were considered statistically significant.

## Results

### Features of the Isolates

A total of 809 *E. coli* isolates from community-acquired adult urinary tract infections (CA-UTIs) were collected during the period 2016–2017. Most infections were lower UTIs (96.8%, 783/809), which included 5 cases of urethritis and 778 cases of cystitis. Upper UTIs accounted for 3.2% (26/809) including eight acute pyelonephritis, four hydronephrosis, seven kidney stones, and seven ureteral calculus. Isolates from female patients accounted for 85% of the total isolates. The ages of patients were as follows: 18–45 years, 31.8%; 46–65 years, 38.8%, and ≥ 66 years, 29.4%.

### *In vitro* Susceptibility of *Escherichia coli* Isolates From Community-Acquired Adult Urinary Tract Infections

Among the 809 *E. coli* isolates studied, 308 (38.07%) were ESBL-producing strains, 477 (58.96%) were ESBL-negative, and 24 (2.97%) were ESBL-uncertain. The antimicrobial agents with susceptibility rates of greater than 95% included imipenem (99.9%), colistin (99.6%), ertapenem (98.9%), amikacin (98.3%), cefmetazole (97.9%), nitrofurantoin (96%), and fosfomycin (95.4%). However, the susceptibility rates to cephalosporins were relatively low, ranging from 58.6 to 74.9%. The antimicrobial susceptibility for all isolates is summarized in Table 2.

**TABLE 2:** The antimicrobial susceptibilities of 809 *Escherichia coli* strains isolated from community-acquired urinary tract infection (CA-UTI) in China.

Antibiotic	All isolates (*n* = 809)						ESBL-producing and ESBL-uncertain isolates (*n* = 332)					
	%R	%I	%S	MIC50 (μg/ml)	MIC90 (μg/ml)	MIC range (μg/ml)	%R	%I	%S	MIC50 (μg/ml)	MIC90 (μg/ml)	MIC range (μg/ml)
Imipenem	0.1		99.9	0.125	0.125	0.03–16	0.3		99.7	0.12	0.25	0.03–16
Colistin	0.4		99.6	0.5	0.5	0.12–8	0.6		99.4	0.5	0.5	0.12–4
Ertapenem	0.2	0.9	98.9	0.016	0.125	0.008–32	0.6	2.1	97.3	0.03	0.25	0.008–32
Amikacin	1.2	0.5	98.3	2	4	0.25–512	3	0.6	96.4	2	8	1–512
Cefmetazole	1.4	0.7	97.9	1	4	0.06–256	2.4	1.5	96.1	1	8	0.06–256
Nitrofurantoin	1.6	2.3	96	16	32	0.5–256	2.7	4.2	93.1	16	32	4–256
Fosfomycin	3.6	1	95.4	1	16	0.25–512	8.4	1.5	90.1	2	64	0.5–512
Cefoperazone/sulbactam	3.5	7.5	89	1	32	0.06–256	8.1	18.1	73.8	16	32	0.06–256
Ceftazidime	20.4	4.7	74.9	0.25	32	0.03–64	49.7	11.4	38.9	8	64	0.12–64
Cefepime	31.5	3.5	65	0.064	64	0.016–64	76.8	8.4	14.8	64	64	0.03–64
Ceftriaxone	38.3	0.2	61.4	0.064	64	0.016–64	93.1	0.6	6.3	64	64	0.016–64
Cefotaxime	38.3	0.4	61.3	0.064	64	0.016–64	93.4	0.9	5.7	64	64	0.03–64
Cefazolin	41.4		58.6	4	32	1–32	93.1		6.9	32	32	1–32
Levofloxacin	50.2	1	48.8	2	16	0.016–64	72.3	0.6	27.1	8	16	0.016–64
Trimethoprim/sulfamethoxazole	55.6		44.4	8	8	0.25–8	68.7		31.3	8	8	0.25–8

**FIGURE 2:**
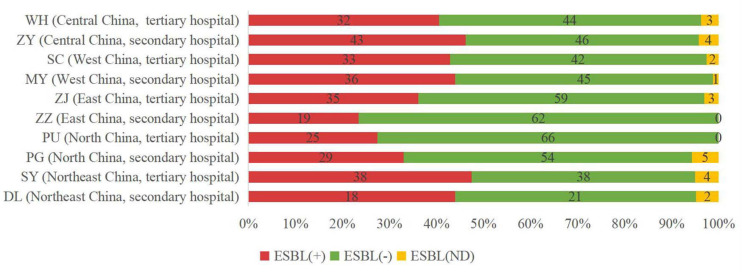
Incidence of extended-spectrum beta-lactamase (ESBL)-producing strains in different sites of China. ESBL (+), ESBL-positive isolates; ESBL (−), ESBL-negative isolates; and ESBL (ND), ESBL-uncertain isolates.

We investigated ESBL differences between isolates from secondary hospitals and tertiary hospitals. Overall, there was no major difference in the proportion of ESBL-positive isolates (37.66% in secondary hospitals vs. 38.44% in tertiary hospitals) or ESBL-uncertain strains (3.12% in secondary hospitals vs. 2.83% in tertiary hospitals) (Figure 2). However, we found some differences, although very small, in the distribution of ESBLs by geographic region. Relatively higher percentages of ESBL rate were found in the northeast (46.3%), central (43.6%), and west (43.4%) China, compared with the sites in the north (30.2%) and east (30.3%) of China (Figure 2).

In general, *E. coli* isolates collected from secondary and tertiary hospitals showed high susceptibility rates to most antibiotics as follows: imipenem (100% vs. 99.8%), colistin (100% vs. 99.3%), ertapenem (99.5% vs. 98.3%), amikacin (98.4% vs. 98.1%), cefmetazole (97.9% vs. 97.9%), fosfomycin (95.8% vs. 95.0%), and nitrofurantoin (94% vs. 97.9%). Furthermore, cefoperazone/sulbactam showed high activity against strains isolated from secondary and tertiary hospitals, with a susceptibility rate of 90.4% and 87.7%. The susceptibilities to cephalosporins in isolates collected from tertiary hospitals varied from 58.0 to 75.5%.

### Characterization of Extended-Spectrum Beta-Lactamase and AmpC Genes

**FIGURE 3:**
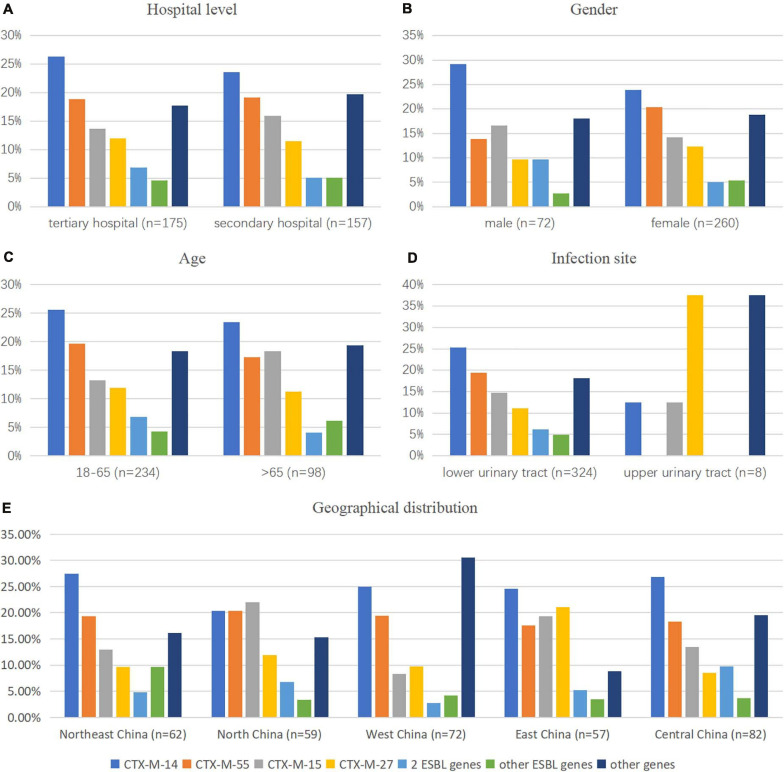
Comparison of the CTX-M gene pattern of *E. coli* isolates by **(A)** hospital category, **(B,C,E)** demographic characteristics, and **(D)** clinical features. *2 ESBL genes*: CTX-M-1 coexisted with CTX-M-9 groups; *other ESBL genes*: genes that existed in less than 10 isolates in this study, such as CTX-M-3 (*n* = 5), CTX-M-64 (*n* = 3), CTX-M-65 (*n* = 9), and CTX-M-79 (*n* = 1); *other genes*: isolates with genes except ESBL genes detected in this study.

Polymerase chain was performed on 332 ESBL-producing and ESBL-uncertain isolates to determine the presence of EBSL and AmpC. The results are shown in Figure 3. The major β-lactamase family detected in the ESBL-producing *E. coli* strains was CTX-M-9 group (149/308, 48.4%), followed by the CTX-M-1 group (136/308, 44.2%). The CTX-M-2 group was not detected. *bla*_*CMY–*__2_ was detected in one isolate. Overall, 7 *bla*_*CTX–M*_ subtypes were detected: *bla*_*CTX–M–*__14_ (98 isolates, 31.8%), *bla*_*CTX–M–*__55_ (72 isolates, 23.4%), *bla*_*CTX–M–*__15_ (54 isolates, 17.5%), *bla*_*CTX–M–*__27_ (41 isolates, 13.3%), *bla*_*CTX–M–*__65_ (10 isolates, 3.2%), *bla*_*CTX–M–*__3_ (5 isolates, 1.6%), *bla*_*CTX–M–*__64_ (4 isolates, 1.3%), and *bla*_*CTX–M–*__79_ (1 isolates, 0.3%). Among the *bla*_*CTX–M*_ subtypes, two different variants were detected in 20/332 isolates (6.0%), most of which were *bla*_*CTX–M–*__14_ and *bla*_*CTX–M–*__15_ (7 isolates), *bla*_*CTX–M–*__14_ and *bla*_*CTX–M–*__55_ (7 isolates).

Among ESBL-uncertain isolates, *bla*_*CMY*_ was the most common AmpC gene (18/24, 75%), consisting of *bla*_*CMY–*__2_ (15 isolates, including 2 isolates coexisting with *bla*_*CTX–M–*__14_), *bla*_*CMY–*__42_ (2 isolates coexisted with *bla*_*CTX–M–*__15_), and *bla*_*CMY–*__34_ (1 isolate). *bla*_*TEM–*__1_ was determined in eight strains (33.3%, 1 isolate coexisted with *bla*_*CTX–M–*__55_). In five strains (20.8%), no ESBL genes were determined.

### *In vitro* Susceptibility of *Escherichia coli* Strains With Different Extended-Spectrum Beta-Lactamase Phenotypes

**FIGURE 4:**
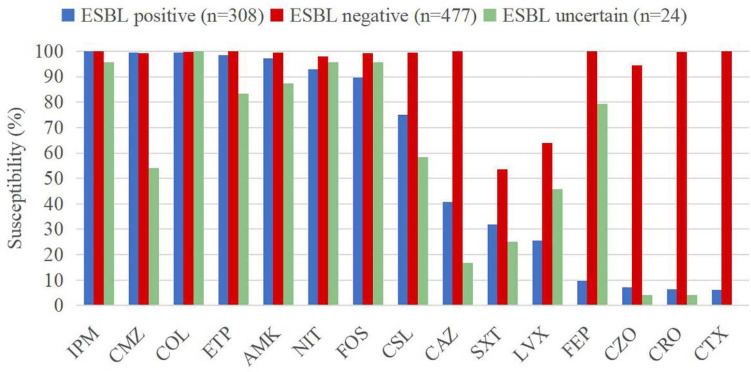
Susceptibility rates of CA-UTI *Escherichia coli* strains with different ESBL phenotypes. IMP, imipenem; ETP, ertapenem; COL, colistin; AMK, amikacin; CMZ, cefmetazole; NIT, nitrofurantoin; FOS, fosfomycin; CSL, cefoperazone/sulbactam; CAZ, ceftazidime; FEP, cefepime; CRO, ceftriaxone; CTX, cefotaxime; CZO, cefazolin; LVX, levofloxacin; and SXT, trimethoprim/sulfamethoxazole.

Extended-spectrum beta-lactamase producing *E. coli* strains exhibited susceptibility rates of over 92% to imipenem (100%), cefmetazole (99.4%), colistin (99.4%), ertapenem (98.4%), amikacin (97.1%), and nitrofurantoin (92.9%). The susceptibility rates of ESBL-negative isolates against all the antimicrobial agents were higher than 90%, except levofloxacin (63.9%) and trimethoprim/sulfamethoxazole (53.5%). On the other hand, ESBL-uncertain isolates showed high susceptibility rates to colistin (100%)], imipenem (95.8%), fosfomycin (95.8%), and nitrofurantoin (95.8%). The susceptibility rate differences between ESBL-producing and ESBL-negative isolates were greater for cephalosporins (Figure 4), including cefotaxime (6.2% vs. 100%), ceftriaxone (6.5% vs. 99.8%), cefazolin (7.1% vs. 94.5%), and cefepime (9.7% vs. 100%).

### Comparison of the Antimicrobial Susceptibility Rates of *Escherichia coli* Isolates by Hospital Level, Demographic Characteristics, and Clinical Features

**FIGURE 5:**
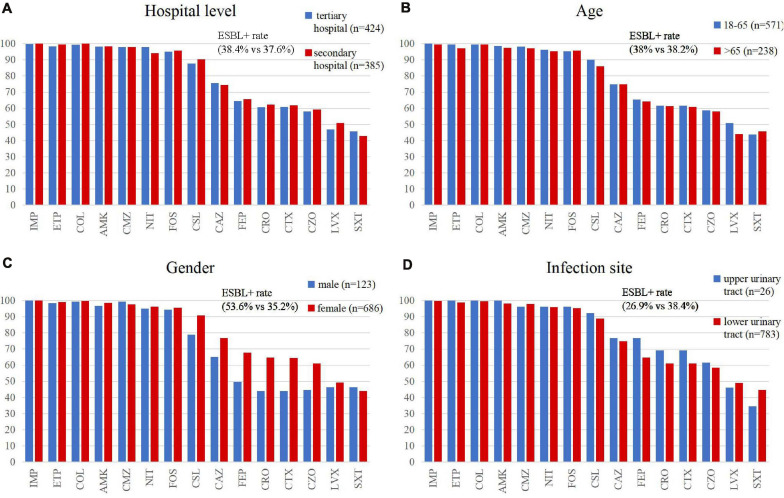
Comparison of the antimicrobial susceptibility rates of *E. coli* isolates by **(A)** hospital category, **(B,C)** demographic characteristics, and **(D)** clinical features. IMP, imipenem; ETP, ertapenem; COL, colistin; AMK, amikacin; CMZ, cefmetazole; NIT, nitrofurantoin; FOS, fosfomycin; CSL, cefoperazone/sulbactam; CAZ, ceftazidime; FEP, cefepime; CRO, ceftriaxone; CTX, cefotaxime; CZO, cefazolin; LVX, levofloxacin; and SXT, trimethoprim/sulfamethoxazole.

We observed that gender was a significant factor influencing antimicrobial susceptibility, with a significantly higher rate of ESBL-producing strains in male (53.6%) than in female patients (35.2%) (*p* < 0.001). Cephalosporins exhibited higher rates of *in vitro* activity against *E. coli* strains from female than from male patients (*p* < 0.05), including cefazolin, ceftazidime, ceftriaxone, cefotaxime, cefoperazone/sulbactam, and cefepime (Figure 5 and Table 3). There was no significant difference in antimicrobial susceptibility and *esbl* genes between *E. coli* strains from tertiary and secondary hospitals, between the age groups of 18 and 65 and over 65 years, or between upper and lower UTIs. The major CTX-M variant in northeast China, west China, and central China was CTX-M-14, followed by CTX-M-15, CTX-M-55, and CTX-M-27. While in north China, the major variant was CTX-M-15 (13/59, 22.03%), in east China, the secondary variant was CTX-M-27 (12/57, 21.05%). The CTX-M genotypes included high rates of CTX-M-14 followed by CTX-M-55, CTX-M-15, and CTX-M-27, which was similar between isolates from tertiary hospitals and from secondary hospitals. There existed a difference in the distribution of CTX-M variants; CTX-M-15 had a larger proportion than CTX-M-55 among male patients and patients over 65 years of age (Figure 3).

**TABLE 3:** Comparison of the antimicrobial susceptibility rates of *E. coli* isolates in hospital level, demographic characteristics, and clinical features.

Antibiotic	Hospital level (%)			Age (%)			Gender (%)			Infection sites (%)		
	Tertiary hospital, *n* = 424	Secondary hospital, *n* = 385	*p*-Value	18–65, *n* = 571	> 65, *n* = 238	*p*-Value	Male, *n* = 123	Female, *n* = 686	*p*-Value	Upper urinary tract, *n* = 26	Lower urinary tract, *n* = 783	*p*-Value
ESBL rate (%)	37.7	35.3	0.985	38	38.2	0.998	53.6	35.2	< 0.001*	26.9	38.4	0.490
Imipenem	99.8	100	1	100	99.6	0.294	100	99.9	1	100	99.9	1
Ertapenem	98.3	99.5	0.153	99.6	97.1	0.005*	98.4	99	0.488	100	98.9	1
Colistin	99.3	100	0.251	99.6	99.6	1	99.2	99.7	0.391	100	99.6	1
Amikacin	98.1	98.4	0.908	98.6	97.5	0.139	96.7	98.5	0.089	100	98.2	1
Cefmetazole	97.9	97.9	1	98.2	97.1	0.440	99.2	97.7	0.871	96.2	98	0.425
Nitrofurantoin	97.9	94	0.017*	96.3	95.4	0.767	95.1	96.2	0.297	96.2	96	0.442
Fosfomycin	95	95.8	0.818	95.3	95.8	0.564	94.3	95.6	0.198	96.2	95.4	0.273
Cefoperazone/sulbactam	87.7	90.4	0.399	90.2	86.1	0.232	78.9	90.8	0.001*	92.3	88.9	1
Ceftazidime	75.5	74.3	0.910	75	74.8	0.954	65	76.7	0.023*	76.9	74.8	0.964
Cefepime	64.4	65.7	0.862	65.3	64.3	0.281	49.6	67.8	< 0.001*	76.9	64.6	0.390
Ceftriaxone	60.6	62.3	0.302	61.5	61.3	0.770	43.9	64.6	< 0.001*	69.2	61.2	0.573
Cefotaxime	60.8	61.8	0.207	61.5	60.9	0.974	43.9	64.4	< 0.001*	69.2	61	0.587
Cefazolin	58	59.2	0.729	58.8	58	0.821	44.7	61.1	0.001*	61.5	58.5	0.756
Levofloxacin	46.9	50.9	0.330	50.8	44.1	0.105	46.3	49.3	0.374	46.2	48.9	0.323
Trimethoprim/sulfamethoxazole	45.8	42.9	0.407	43.8	45.8	0.599	46.3	44	0.634	34.6	44.7	0.309

*p < 0.05.

### Community-Acquired Carbapenem-Resistant Strains

In this study, two strains were identified as community-acquired carbapenem resistant. The two carbapenem-resistant *Enterobacteriaceae* (CRE) strains (MYU26 and SYU04) were resistant to almost all β-lactam antibiotics tested, with only three antimicrobial agents exhibiting potent activity against them, including colistin (MYU26: MIC ≤ 0.12, SYU04: MIC = 0.5), fosfomycin (MYU26: MIC = 1, SYU04: MIC = 4), and tigecycline (MYU26 and SYU04: MICs ≤ 0.06). MYU26 carried the *bla*_*CTX–M–*__15_ and *bla*_*CMY–*__42_ genes and SYU04 carried the *bla*_*CMY–*__2_ gene. No carbapenemase genes were detected.

## Discussion

Our analysis of stringently selected *E. coli* isolates from CA-UTIs, collected in China during the period 2016–2017, revealed a relatively lower ESBL rate (38.07%) than previously reported in 2012 (68.6%) and 2014 (59.1%) in a study from The Study for Monitoring Antimicrobial Resistance Trends (SMART) surveillance program (Yang et al., 2017). However, another study in China (Zhang et al., 2019) found no significant difference in the rates of ESBLs among *E. coli* isolates from HA and CA UTIs, and the ESBL rate in that study was 48.8% among CA-UTI *E. coli* isolates during the period 2016–2017, which is higher than in the present study. In Asia, the proportion of ESBL-producing *E. coli* isolates was 4.1% in patients with acute uncomplicated cystitis in Japan (Hayami et al., 2019), with 10.8% rates in CA-UTI *E. coli* isolates in Korea (Park et al., 2017). In Europe, the frequency of ESBL-producing CA-UTI *E. coli* strains ranged from 2.2 to 24% (Yılmaz et al., 2016; Chervet et al., 2018; van Driel et al., 2019). In North America, the rates varied from 14.1% in Canada and 16.0% in the UnitedStates to 31.3% in Mexico (Lob et al., 2016; Galindo-Méndez, 2018). The difference in the prevalence of ESBLs among different studies may be due to population source of the isolates.

Our results show that CTX-M-14, CTX-M-55, and CTX-M-15 are the most dominant CTX-M variants in the ESBL-producing *E. coli*, followed by CTX-M-27, CTX-M-3, CTX-M-65, CTX-M-64, and CTX-M-79. CMY-2 is the major β-lactamase in the ESBL-uncertain *E. coli*. In different regions, the CTX-M variant pattern is different. In north China, CTX-M-15 (22.03%), CTX-M-14, and CTX-M-55 (20.34%) were distributed equally in the ESBL-producing and uncertain (phenotype) isolates, while in other regions, CTX-M-14 was the major variant with over 24% rate, and the rates of CTX-M-15 and CTX-M-55 were less than 20%. In east China, CTX-M-27 (21.05%) was the secondary variant that exceeded CTX-M-15 (19.3%) and CTX-M-55 (17.54%). In addition, another report also shows that CTX-M-27 has become more prevalent in East and Southeast Asia (Chong et al., 2018). Among the isolates from lower urinary tract infections, *bla*_*CTX–M–*__14_ was the most common ESBL gene type, which was different from upper urinary tract infections (the main gene was *bla*_*CTX–M–*__27_), but it may not be comprehensive since the number of isolates was extremely low in upper urinary tract infections (*n* = 8).

Most of CTX-Ms, such as CTX-M-14, CTX-M-3, and CTX-M-65, exhibit powerful activity against cefotaxime and ceftriaxone but not ceftazidime. Some CTX-Ms, such as CTX-M-15, CTX-M-27, CTX-M-55, and CTX-M-64, exhibit enhanced catalytic efficiencies against ceftazidime (Zhao and Hu, 2013). In our study, the rate of ceftazidime-resistant isolates (49.7%) corresponds to the rate of isolates that carried CTX-M-15/55/27 (48.8%). In addition, among the ESBL-producing and uncertain (phenotype) isolates, the rates of ceftriaxone/cefotaxime/cefazolin-resistant were 93.1%, 93.4%, and 93.1%, respectively. Although CTX-M can be inhibited by β-lactamase inhibitors as sulbactam, clavulanate, and tazobactam, the susceptibility rate of cefoperazone/sulbactam was only 73.8%. Moreover, most of ESBL-uncertain isolates carried CMY-2, which cannot be inhibited by β-lactamase inhibitors. For these isolates, fosfomycin would be useful for the empirical treatment of acute cystitis since it had high rates of activity, with a susceptibility rate of over 90%.

The common antimicrobial drugs for treating acute uncomplicated cystitis include fosfomycin, nitrofurantoin, trimethoprim/sulfamethoxazole, and β-lactams, including cephalexin, cefaclor, and amoxicillin/clavulanate. Besides, β-lactams and quinolones are recommended as renal excretion-type antibiotics for acute uncomplicated pyelonephritis (Choe et al., 2018). However, the two old antibiotics, fosfomycin and nitrofurantoin, which achieve high urinary concentrations and minimal toxicity, are not often used in China (Qiao et al., 2013), yet *E. coli* accounts for the majority of pathogens causing CA-UTIs (Qiao et al., 2013; Yang et al., 2017). It is important to understand the activity of β-lactams and quinolones against *E. coli* strains to guide empirical antimicrobial therapy decision making.

In the European Association of Urology guidelines updated in 2020, cephalosporins are recommended for oral empirical treatment of uncomplicated pyelonephritis and as alternative antimicrobials for therapy in uncomplicated cystitis (Bonkat et al., 2020). However, in the present study, cephalosporins had poor activity against ESBL-producing *E. coli* strains. Susceptibility rates of *E. coli* to the first-, third-, and fourth-generation cephalosporins were lower than 80%, among which, the susceptibility rates were 76.9% (for strains that caused pyelonephritis) and 74.8% (for strains that caused lower urinary tract infections) for ceftazidime, 69.2% and 61% for cefotaxime, 69.2% and 61.2% for ceftriaxone, and 76.9% and 64.6% for cefepime, respectively, which indicate that these agents might not be the optimum medications for empirical UTI therapies.

In the present study, the proportion of ESBL-non-producing *E. coli* isolates was 73.1% in pyelonephritis and 61.6% in lower UTIs, which is consistent with the cephalosporin susceptibility rates. Genes that encode for ESBLs are usually found on large plasmids accompanied by genetic determinants of resistance against multiple classes of antibiotics, such as aminoglycosides, sulfonamides, and fluoroquinolones (Lee et al., 2012; Bader et al., 2017). Our study also investigated the differences in ESBL carriage rates and cephalosporin susceptibility rates between males and females. Significantly higher ESBL and cephalosporin resistance rates were found in *E. coli* isolates from men. ESBL rates range from 30.2% to 46.3% in different regions of China, with northeast, central, and west China having higher rates in *E. coli*. The choice of cephalosporins for treatment of UTIs should be based on local prevalence of ESBL-producing isolate data.

Fluoroquinolones and cephalosporins are antimicrobial agents that can be recommended for oral empirical treatment of uncomplicated pyelonephritis. Meanwhile, ciprofloxacin, levofloxacin, and ofloxacin are not recommended in the treatment of uncomplicated cystitis. However, quinolone use has been compromised by the high resistance rates to most bacterial pathogens (Kim and Hooper, 2014), with a 50.2% resistance rate reported in this study higher than the rates reported in patients with acute uncomplicated cystitis in Japan (6.4%) (Yılmaz et al., 2016). Quinolone resistance mechanisms are multiple and complicated. Chromosomal gene mutation including *gyrA*, *gyrB*, *parC*, and *parE* genes, reduce binding of the drug to the enzyme–DNA complex. Furthermore, overexpression of native efflux pumps localized in the bacterial membrane may cause resistance to quinolones and other antimicrobials. Additionally, plasmid-mediated quinolone resistance determinants can encode additional antimicrobial resistances and transfer multidrug resistance to a variety of antimicrobials, including quinolones (Hooper and Jacoby, 2015). Through these resistance mechanisms, the number of quinolone-resistant bacterial strains has grown steadily over the years. A previous study reported that S83L/D87N in *gyrA* and S80I in *parC* were the most common topoisomerase mutations in ESBL-producing *E. coli* isolates from the community (Ni et al., 2016). In addition, the majority of UTIs in the present study were uncomplicated cystitis (96%, 778/809) for which fluoroquinolones are not recommended for treatment. Thus, in this case, the use of fluoroquinolones for empiric treatment of UTIs should be restricted.

Although carbapenems are not recommended as the first-line treatment for uncomplicated cystitis and pyelonephritis (Bonkat et al., 2020), the carbapenems exhibited high *in vitro* activity against ESBL-producing *E. coli* in the present study, with a susceptibility rate of 100% for imipenem and 98.4% for ertapenem. Given the relatively high ESBL rates in CA-UTIs in the present study, and the low susceptibility rates to beta-lactam and fluoroquinolones, carbapenems can be considered for empiric therapy in patients with suspected ESBL-producing and multidrug-resistant (MDR) bacterial strains (Essack, 2000; Paterson, 2000; Livermore et al., 2001). On the other hand, in order to maintain the activity of carbapenems, it is necessary to replace carbapenems by other antimicrobials once the susceptibilities to antimicrobial agents have been confirmed. Carbapenem-resistant *E. coli* (CREc) constituted 0.25% (2/809) of the CA-UTI *E. coli* isolates studied, which is consistent with previous reports. Findings from previous studies indicate an increase in the prevalence of community-acquired CRE in Taiwan (Lai et al., 2013; Tang et al., 2016). CA-CREs have also been described in the southeastern part of the United States (Thaden et al., 2014), suggesting widespread distribution of the organism in the community, and patients infected with CA-CRE have more urinary tract infections (Tang et al., 2016). The two patients with CREc were both elderly and female, which is in agreement with previous findings (Tang et al., 2016). In this study, amikacin colistin, fosfomycin, and tigecycline exhibited potent activity against CREs, suggesting that colistin, fosfomycin, tigecycline, and aminoglycosides could be treatment choices for UTIs caused by CRE (Bader et al., 2017).

As with majority of the studies, a limitation in this study was the lack of genomic analysis such as multilocus sequence typing (MLST). Since all the isolates were collected from 10 different regions in China and the number per site was no more than 100, there was a high possibility that these isolates were sporadic and rarely clonal outbreak. From other studies, we learned that ST131, ST69, ST95, and ST73 were the dominant sequence types (STs) in *E. coli* isolated from urinary tract infections (Riley, 2014; Yamaji et al., 2018; Kot, 2019). Among multidrug-resistance *E. coli* isolates associated with CA-UTIs, ST131 and ST69 were predominant in Australia and Saudi Arabia (Alghoribi et al., 2015; Rogers et al., 2015); however, ST648, ST224, ST38, and ST405 also occurred in China (Cao et al., 2011, 2014).

This study also found that two of three colistin-resistant *E. coli* isolates carried *bla_*CTX–M–*__14_* and *bla_*TEM–*__1_* genes but were susceptible to carbapenems, which is consistent with a previous study (Jiang et al., 2020). The results suggest we need to attach great importance to the management of MDR Gram-negative bacteria. This study showed the real ESBL rates and genotype distribution in community-acquired UTIs through strict selection criteria, since the strategies for treatment of hospital- and community-acquired UTIs are different. Besides, some UTIs might not represent genuine community acquisition if the patients were admitted to a hospital before infection. Hence, this strictly defined clinical epidemiological study in CA-UTIs will help the clinicians to better understand the antimicrobial resistance status and select empiric antimicrobial agents.

## Conclusion

Our findings show that ESBLs are still a significant issue in *E. coli* isolates from CA-UTI in China, with an average prevalence of 38.07%. Higher rates of ESBL among the *E. coli* strains were confined to the northeast, central, and west parts of China. The choice of antimicrobial agents for the treatment of CA-UTIs should be based on local surveillance data. Use of fluoroquinolones for empiric treatment of UTIs should be restricted due to high resistance rate. Carbapenems can be used empirically for highly suspected ESBL-producing and MDR strains. However, the occurrence of CRE in the community is a cause for concern.

## Data Availability Statement

The original contributions presented in the study are included in the article/supplementary material, further inquiries can be directed to the corresponding author/s.

## Ethics Statement

The protocol has been reviewed by the human research Ethics Committee of the Institutional Review Board (IRB) of the Peking Union Medical College Hospital (Ethics Approval Number: S-K136). This project did not involve any patient information nor did it affect the normal diagnosis and treatment of patients, and after consultation with the IRB, formal ethical approval was reviewed and waived, and written patient consent was not required.

## Author Contributions

QY conceived and designed the experiments. YY, GZ, and JZ performed the experiments. PJ, XL, and YZ analyzed the data and wrote the manuscript. TK, YX, and QY reviewed the manuscript and polished the language. All authors read and approved the final manuscript.

## Conflict of Interest

The authors declare that the research was conducted in the absence of any commercial or financial relationships that could be construed as a potential conflict of interest.

## Footnotes

1 http://blast.ncbi.nlm.nih.gov/Blast.cgi
